# *In vivo* Proximity Labeling of Nuclear and Nucleolar Proteins by a Stably Expressed, DNA Damage-Responsive NONO-APEX2 Fusion Protein

**DOI:** 10.3389/fmolb.2022.914873

**Published:** 2022-06-06

**Authors:** Barbara Trifault, Victoria Mamontova, Kaspar Burger

**Affiliations:** ^1^ Mildred Scheel Early Career Center for Cancer Research (Mildred-Scheel-Nachwuchszentrum, MSNZ), University Hospital Würzburg, Würzburg, Germany; ^2^ Department of Biochemistry and Molecular Biology, Biocenter of the University of Würzburg, Würzburg, Germany

**Keywords:** APEX2, proximity labeling, NONO, paraspeckles, nucleolus, DNA damage

## Abstract

Cellular stress can induce DNA lesions that threaten the stability of genes. The DNA damage response (DDR) recognises and repairs broken DNA to maintain genome stability. Intriguingly, components of nuclear paraspeckles like the non-POU domain containing octamer-binding protein (NONO) participate in the repair of DNA double-strand breaks (DSBs). NONO is a multifunctional RNA-binding protein (RBP) that facilitates the retention and editing of messenger (m)RNA as well as pre-mRNA processing. However, the role of NONO in the DDR is poorly understood. Here, we establish a novel human U2OS cell line that expresses NONO fused to the engineered ascorbate peroxidase 2 (U2OS:NONO-APEX2-HA). We show that NONO-APEX2-HA accumulates in the nucleolus in response to DNA damage. Combining viability assays, subcellular localisation studies, coimmunoprecipitation experiments and *in vivo* proximity labeling, we demonstrate that NONO-APEX2-HA is a stably expressed fusion protein that mimics endogenous NONO in terms of expression, localisation and *bona fide* interactors. We propose that *in vivo* proximity labeling in U2OS:NONO-APEX2-HA cells is capable for the assessment of NONO interactomes by downstream assays. U2OS:NONO-APEX2-HA cells will likely be a valuable resource for the investigation of NONO interactome dynamics in response to DNA damage and other stimuli.

## 1 Introduction

The synthesis of mRNA correlates with the number of proteins and is buffered against changes in gene copy number (Crissman and Steinkamp, 1973; Padovan-Merhar et al., 2015; Voichek et al., 2016). To maintain homeostasis, gene expression is sensitive to cellular stress including DNA damage. The response to DSBs engages kinases and chromatin-modifying enzymes to restrict the accessibility of the RNA synthesis machinery to DSBs and transiently silence transcription (Blackford and Jackson, 2017; Burger et al., 2019a; Jackson and Bartek, 2009; Kruhlak et al., 2007; Price and D’Andrea, 2013). The DDR also targets numerous RBPs to mitigate the RNA metabolism (Matsuoka et al., 2007; Smolka et al., 2007; Adamson et al., 2012; Bader et al., 2020; Klaric et al., 2021). Thus, spurious mRNA synthesis is potentially hazardous and leads to aberrant transcripts that interfere with DSB repair (Aguilera and Gómez-González, 2008; Caron et al., 2019; Machour and Ayoub, 2020). However, the inhibition of transcription on broken DNA is incomplete and nascent RNA can serve as template for repair (Storici et al., 2007; Chakraborty et al., 2016; Iannelli et al., 2017). DSBs trigger the *de novo* transcription and accumulation of small non-coding DNA damage response RNA (DDRNA), which promotes the efficient recruitment of DSB repair factors (Francia et al., 2012; Wei et al., 2012; Francia et al., 2016; Wang and Goldstein, 2016; Burger et al., 2017; Michelini et al., 2017; Burger et al., 2019b). Thus, the RNA metabolism and RBPs emerge as critical regulators of genome stability and potential effectors of DSB repair (Chowdhury et al., 2013; Michelini et al., 2018; Zong et al., 2020).

NONO functions in numerous RNA metabolic processes and is frequently deregulated in cancer (Shav-Tal and Zipori, 2002; Knott et al., 2016; Feng et al., 2020). NONO enriches in nuclear RNA-protein condensates called paraspeckles to retain a subset of pre-mRNA for editing (Zhang and Carmichael, 2001; Prasanth et al., 2005; Bond and Fox, 2009). On chromatin, NONO modulates the initiation, elongation and termination of RNA polymerase II (RNAPII) (Basu et al., 1997; Emili et al., 2002; Kameoka et al., 2004; Amelio et al., 2007; Kaneko et al., 2007; Ma et al., 2016; Li et al., 2021). Intriguingly, NONO is linked to genome maintenance. The depletion of NONO causes defects in DSB repair and instable telomeres (Li et al., 2014; Petti et al., 2019). NONO accumulates at UV lesions and linearised DNA ends to promote DSB repair (Bladen et al., 2005; Salton et al., 2010; Rajesh et al., 2011; Krietsch et al., 2012; Jaafar et al., 2017). However, the precise role of NONO in the DDR is poorly understood. The multifunctionality of NONO suggests a complex engagement in the DDR and demands new analytic tools. Here, we report a human U2OS cell line that expresses NONO fused to the engineered ascorbate peroxidase 2 (APEX2). We show that NONO-APEX2-HA is a stably expressed, DNA damage-responsive fusion protein that associates with *bona fide* interactors and specifically biotinylates them. Our data suggest that U2OS:NONO-APEX2-HA cells may be a powerful tool to investigate NONO interactomes.

## 2 Materials and Methods

### 2.1 Tissue Culture, Inhibitors, Transfection and Proximity Labeling

Human U2OS and HEK293 cells were cultured in Dulbecco’s modified eagle’s medium (DMEM, Gibco), containing 10% fetal bovine serum (FBS, Capricorn), 100 U/ml penicillin-streptomycin and 2 mM glutamine (Gibco) at 37 °C and 5% CO_2_. Acetic acid (Sigma), etoposide (Sigma) and CX-5461 (Selleckchem) were used as indicated. Small interfering (si)RNA (siControl scrambled, D-001810-01-05; siNONO, L-007756-01-0005, Dharmacon) were transfected with Lipofectamine 2000 (Invitrogen) and serum-reduced medium (OptiMEM, Gibco) according to the manufacturer’s protocol. For proximity labeling cells were incubated with 0.5 mM biotin-phenol (Iris Biotech) for 30 min at 37 °C and 1 mM hydrogen peroxide (Sigma). Labeling was quenched by 10 mM sodium ascorbate (Sigma), 5 mM trolox (Sigma) and 10 mM sodium azide (Sigma).

### 2.2 Cloning

Coding sequences were PCR-amplified from pcDNA3.1-APEX2-NES (Addgene) and pmCherry-NONO (Ling-Ling Chen, Shanghai Institute of Biochemistry and Cell Biology). To create pRRL-APEX2-HA, amplified APEX2-HA and the pRRL-puro vector (Elmar Wolf, Biocenter Würzburg) were restricted with PacI/SpeI (4 h, 37 °C) and ligated with T4 DNA ligase (16 °C, overnight). To create pRRL-NONO-APEX2-HA, amplified NONO and pRRL-APEX2-HA were digested with PacI/MluI (4 h, 37 °C) and ligated with T4 DNA ligase (16°C, overnight). pRRL-NONO-APEX2-HA was transformed into *E. coli* and purified using a Miniprep Kit (NEB) according to the manufacturer’s protocol. The plasmid was incubated with PacI/SpeI or buffer (4 h, 37°C), separated on a 0.9% agarose 1x TBE gel (20 min, 150 V), and stained with ethidium bromide (0.2 μg/ml, Sigma) under UV light. All enzymes were purchased from NEB. For primers see Supplementary Table S1.

### 2.3 Viral Work

Plasmids (10 µg pRRL-NONO-APEX2-HA, 10 µg pPAX2, 2.5 µg pMD2.G) and 30 µL polyethylenimine (PEI, Calbiochem) were diluted in 500 µL OptiMEM, vortexed, incubated (25 min, RT) and added dropwise to HEK293 cells, which were preincubated in 5 ml DMEM/2% FBS, and transfected for 8 h. Virus was harvested 3x every 12 h, sterile filtered and frozen (-80°C). For infection, U2OS cells were cultured in viral mixture [(1.5 ml DMEM, 1.5 ml pRRL-NONO-APEX2-HA virus, 6 µL polybrene (Invitrogen)] and incubated (24 h, 37°C). The mixture was replaced by DMEM containing 2 μg/ml puromycin (Invivogen) for 10 days of polyclonal selection.

### 2.4 Crystal Violet Staining

Cells were seeded at different densities and incubated for 72 h, washed in PBS, stained in 0.5% crystal violet solution/20% methanol (10 min, RT) and washed (3 × 20 min) in PBS. Stainings were scanned (Epson) and quantified (ImageJ, NIH).

### 2.5 Fluorescence-Activated Cell Sorting

Cells were washed in PBS, trypsinised, resuspended in DMEM and centrifuged (1200 rpm, 3 min, 4°C). Pellets were washed in PBS, centrifuged (1200 rpm, 3 min, 4°C), resuspended in 1 ml PBS and fixed in 4 ml 100% ethanol (−20°C, overnight). Cells were pelleted (1500 rpm, 10 min) and resuspended in 1 ml PBS. 1 x 10^6^ cells were stained with 54 µM propidium iodide (Sigma) in the presence of 24 μg/ml RNase A (Sigma) (30 min, RT, dark), sorted and analysed by a FACSDiva 9.0.1 flow cytometer and software (BD Biosciences).

### 2.6 Immunoblotting and Immunoprecipitation

Cells were lysed, boiled and sonicated in sample buffer (250 mM Tris-HCl pH6.8, 8% SDS, 40% glycerol, 8% β-mercaptoethanol, 0.02% bromophenol blue). Extracts were separated by SDS-PAGE, transferred onto nitrocellulose membranes (GE Healthcare), blocked in PBS/0.1% Triton-X-100/5% milk (PBST) (1 h, RT) and probed with primary antibodies (4°C, overnight). Membranes were washed in PBST, incubated with secondary antibodies (1 h, RT) and washed in PBST without milk. Signals were visualised with an ECL kit (GE Healthcare) and an imaging station (Fuji). Membranes were stained with ponceau S (0.5% ponceau S, 1% acetic acid) prior to blocking. For immunoprecipitation (IP), cells were trypsinised, washed with cold PBS and centrifuged (1200 rpm, 3 min). Pellets were lysed in five volumes IP buffer (200 mM NaCl, 0.5 mM EDTA, 20 mM HEPES, 0.2% NP-40, 10% glycerol, 400U RNase inhibitor, 1 x protease/phosphatase inhibitor) for 10 min on ice. Lysates were centrifuged (13,000 rpm, 10 min) and supernatants were incubated (3 h, 4°C) with 2 µg primary antibodies pre-conjugated with 25 µL Dynabeads (protein G or streptavidin C1, Invitrogen). Immunocomplexes were immobilised on a magnet (Invitrogen), washed with 800 µL IP buffer (3 × 10 min, 4 °C) and eluted with sample buffer (5 min, 95 °C). Gels were silver stained with a kit (Invitrogen) according to the manufacturer’s protocol. For antibodies see Supplementary Table S2.

### 2.7 RNA Immunoprecipitation

Cells were harvested, washed in cold PBS, incubated in five volumes IP buffer (10 min on ice) and centrifuged (10 min, 13,000 rpm). Total RNA from 25% of lysate (input) was resuspended in TRIzol (Invitrogen). Remaining supernatant was split into 25% aliquots and incubated with 4 µg antibodies (3 h, 4°C). Antibodies were pre-conjugated with 25 µL Dynabeads overnight (protein G, Invitrogen). Immune complexes were washed 4 x in 800 µL IP buffer. Total and immunoselected RNA samples were purified using TRIzol according to the manufacturer’s protocol (Invitrogen). For quantitative analysis, samples were reverse-transcribed with Superscript reverse transcriptase III (Invitrogen) using region-specific primers. cDNA was quantified by real-time quantitative PCR using the SensiFAST SYBR No-ROX Kit (Bioline) and a StepOne Real Time PCR thermal cycler (Applied Biosystems). For the calculation of % of input the average cT values measured for input, IP and IgG controls were transformed into absolute replicative concentrations. Formula: conc_abs_ = 1/2^cT. Absolute replicative concentrations of IP and IgG samples were then transformed to relative replicative concentrations by normalising to inputs values, with input values set to 1. Formula: conc_rel_IP = conc_abs_IP/conc_abs_input and conc_rel_IgG = conc_abs_IgG/conc_abs_input. The % of input values were calculated by subtraction of relative IgG values from relative IP values, division by 1 (i.e. relative input values) and multiplication by 100. Formula: [(conc_rel_IP-conc_rel_IgG)/1]*100. For primers see Supplementary Table S1.

### 2.8 Confocal Microscopy

Cells were grown on coverslips, washed with PBS, fixed with 3% para-formaldehyde (Sigma) for 8 min at RT, washed with PBS, permeabilised with PBST (4 min, RT) and blocked in PBS/10% FBS (2 h, 4°C). Primary antibodies were incubated overnight at 4°C in PBS/0.15% FBS. Cells were washed in PBST, incubated with secondary antibodies in PBS/0.15% FBS (1 h, RT) in a dark humidified chamber and washed in PBST. Nuclei were stained with 4‘,6-diamidino-2-phenylindol (DAPI)-containing mounting medium (Vectashield), imaged (CLSM-Leica-SP2) and processed (ImageJ, NIH). Channels were acquired sequentially, between frames, and with equal exposure times. >100 cells per condition were quantified. Colocalisation was assessed by using RGB profiler (ImageJ) and by calculation of the Pearson’s correlation coefficient with JACoP (ImageJ). Parameters were set as described (Bolte and Cordelières, 2006). For antibodies see Supplementary Table S2.

## 3 Results

### 3.1 Generation of U2OS:NONO-APEX2-HA Cells

**FIGURE 1:**
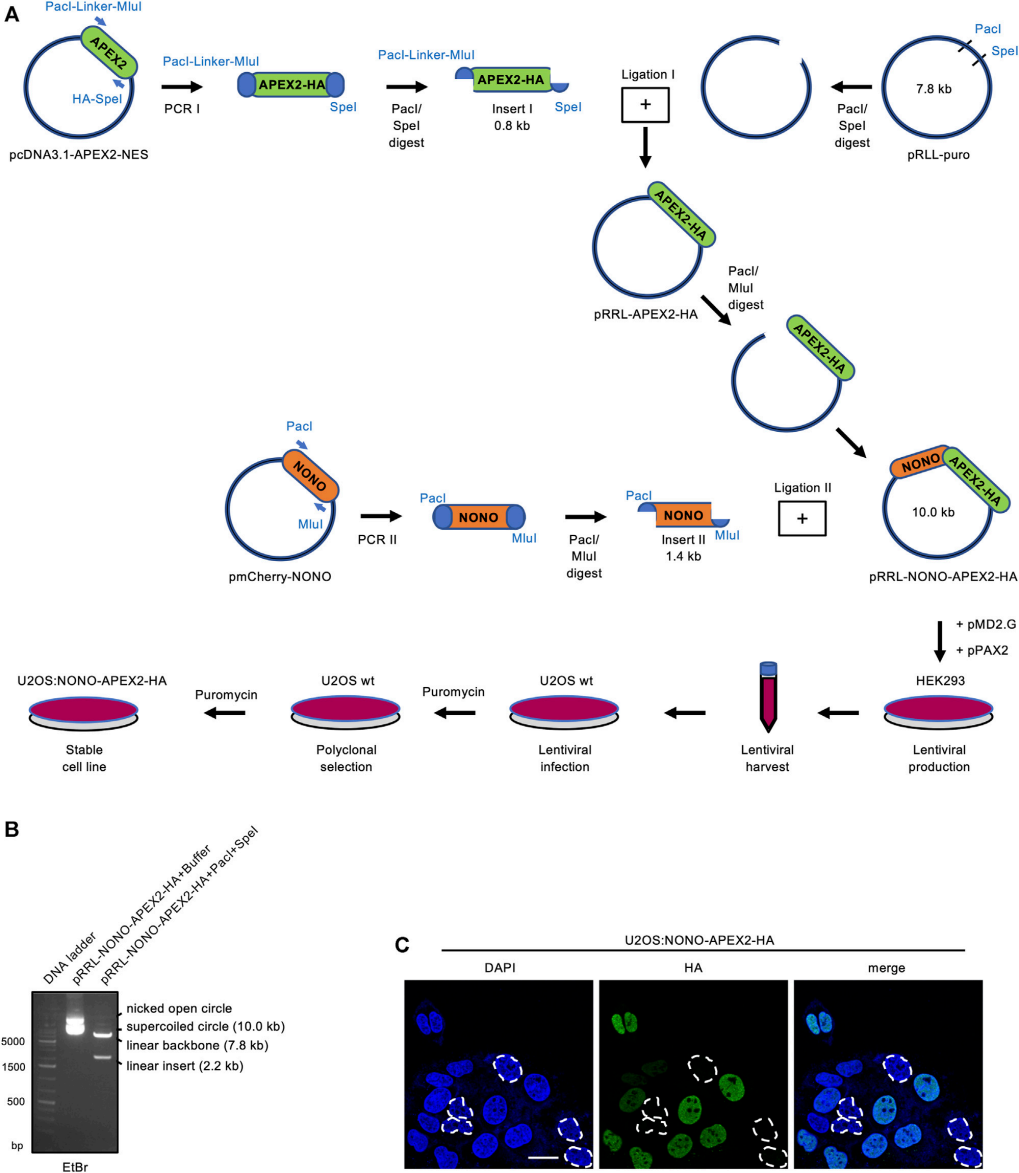
Vector construction and expression of NONO-APEX2-HA. **(A)** Generation of the pRRL-NONO-APEX2-HA vector for lentiviral transduction, polyclonal selection and stable expression in U2OS cells. **(B)** Agarose gel electrophoresis of non-restricted pRRL-NONO-APEX2-HA vector (buffer) or upon incubation with PacI/SpeI. DNA was stained with ethidium bromide (EtBr). **(C)** Confocal imaging of NONO-APEX2-HA. Broken white circles indicate nuclei without substantial NONO-APEX2-HA expression. Representative cells are shown. DAPI, 4‘,6-diamidino-2-phenylindol; scale bar, 10 µm.

We aimed to establish a system that allows the unbiased and stringent detection of NONO interactors by APEX2-mediated *in vivo* proximity labeling. Using PCR cloning, we generated inserts encoding NONO and HA-tagged APEX2, and ligated them into the pRRL expression vector (Figure 1A). An analytic digest displayed the NONO-APEX2-HA insert at the predicted size of 2.2 kb (Figure 1B). Next, we transduced the pRRL-NONO-APEX2-HA construct into U2OS cells and selected a polyclonal U2OS:NONO-APEX2-HA cell line. We used an HA antibody in confocal imaging to determine how many cells express NONO-APEX2-HA and reproducibly scored around 80% HA-positive nuclei in a total number of 280 analysed cells (Figure 1C). We assessed the growth and cell cycle of U2OS:NONO-APEX2-HA cells and observed no significant differences compared to parental cells (Supplementary Figures S1A, S1B). We conclude that the stable expression of NONO-APEX2-HA is non-toxic in U2OS cells.

### 3.2 DNA Damage Induces Accumulation of Endogenous NONO in the Nucleolus

**FIGURE 2:**
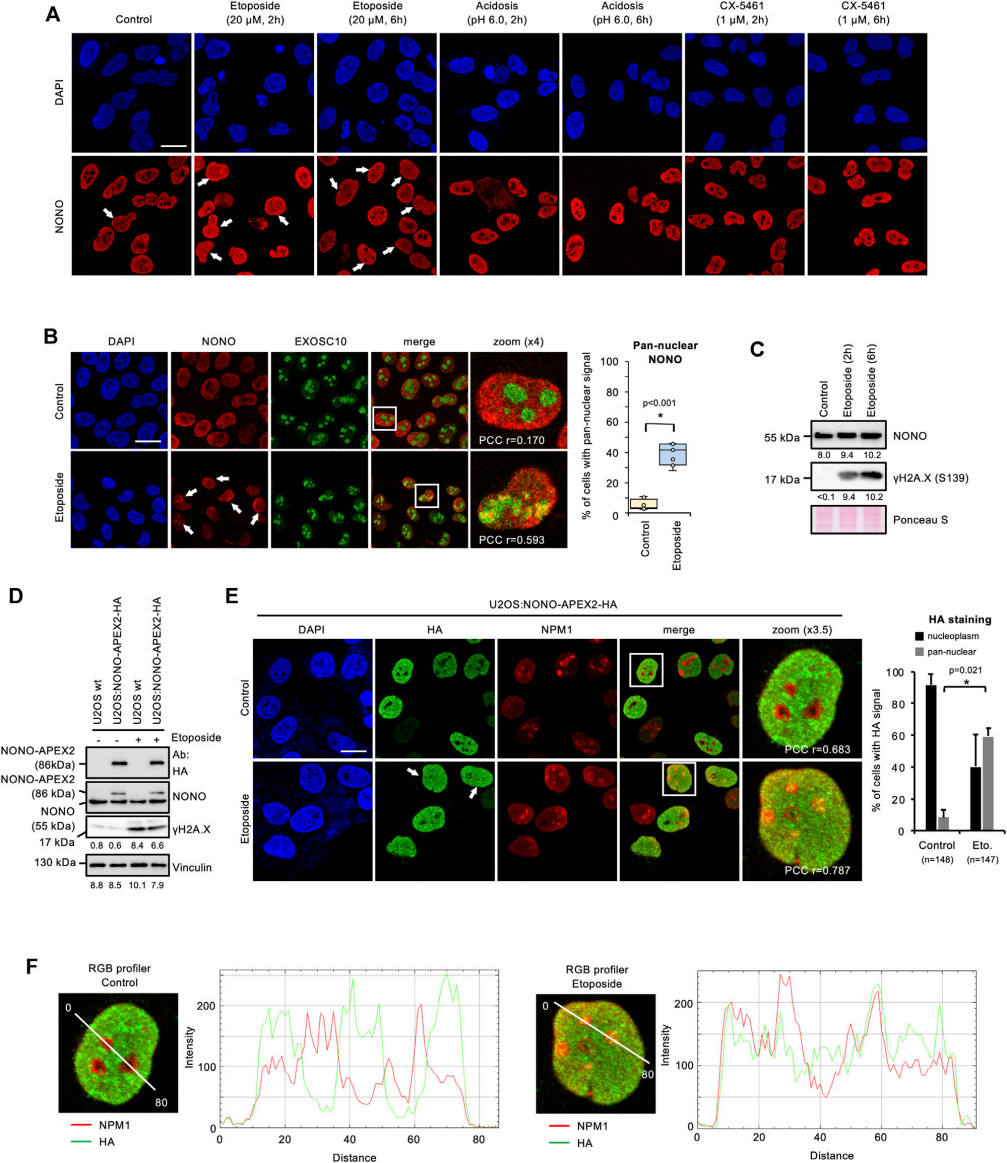
Accumulation of NONO and NONO-APEX2-HA in the nucleolus in response to DNA damage in U2OS cells. **(A)** Confocal imaging of NONO in unperturbed cells (control) or upon incubation with Etoposide, acetic acid (acidosis), or RNA polymerase I inhibitor (CX-5461). **(B)** Confocal imaging of NONO and exosomal subunit EXOSC10 with or without (+/-) Etoposide (20 μM, 2 h) (left). Quantitation of cells with pan-nuclear NONO staining (right). **(C)** Immunoblots detecting NONO and Ser139-phosphorylated histone variant H2A.X (γH2A.X) +/- Etoposide (20 μM, 2 h). **(D)** Immunoblots detecting NONO-APEX2-HA, NONO and γH2A.X ± Etoposide (20 μM, 2 h). Immunoblots were quantified by ImageJ. Signals are shown as arbitrary units. wt, wild type; Vinculin, Ponceau S, loading controls; Ab, antibody. **(E)** Confocal imaging of NONO-APEX2-HA and NPM1 ± Etoposide (20 μM, 2 h) (left). Quantitation of cells with nucleoplasmic and pan-nuclear HA staining (right). **(F)** RGB profiler plots for zoomed panels from **(E)**. Representative cells are shown. DAPI, 4‘,6-diamidino-2-phenylindol; scale bar, 10 μm; white boxes, magnified areas; white arrowheads, pan-nuclear NONO staining; asterisk, *p* < 0.001 (Student’s t-test); n.s., not significant; r = Pearson’s correlation coefficient (PCC).

To analyse the expression and subcellular localisation of NONO, we acquired a specific antibody and first validated its applicability in immune assays by using siRNA technology (Supplementary Figures S2A, S2B). We observed a significant reduction in NONO signals on immunoblots and in confocal imaging upon transfection of NONO-targeting siRNA. NONO accumulates in the nucleolus of DNA-damaged cells (Moore et al., 2011). We incubated U2OS cells with the DNA topoisomerase-II inhibitor etoposide and assessed the localisation of NONO by confocal imaging. We observed pan-nuclear signals for NONO in a subset of cells upon incubation with etoposide, but no other treatments (Figure 2A). We confirmed the DNA damage-induced nucleolar accumulation of NONO by costaining with the nucleolar exosomal subunit EXOSC10 and quantified 30–50% of cells with pan-nuclear NONO staining (Figure 2B and Supplementary Figure S2C), which was further confirmed by RGB profiler measurements (Supplementary Figure S2D). Etoposide also induced partial colocalisation of NONO with nucleolar Nucleophosmin 1 (NPM1) (Supplementary Figure S2E). A fraction of EXOSC10 translocates to the nucleoplasm in response to DNA damage (Domingo-Prim et al., 2019). However, we could not detect prominent colocalisation of EXOSC10 with γH2A.X foci upon incubation with etoposide (Supplementary Figure S3A). Instead, the bulk of EXOSC10 remained nucleolar (Supplementary Figure S3B). We conclude that short-term incubation with etoposide triggers the nucleolar accumulation of NONO and does not impair nucleolar integrity. In response to UV irradiation, the ubiquitin-ligase ring finger protein 8 (RNF8) targets NONO for proteasomal degradation (Deshar et al., 2019). Thus, we monitored the NONO protein level by immunoblotting. Incubation with etoposide strongly induced the serine-139-phosphorylated H2A.X variant (γH2A.X), a marker for DNA damage, but did not alter NONO levels (Figure 2C). NONO binds the I-SceI cleavage site of a DSB reporter (Krietsch et al., 2012). To test if NONO accumulates at endogenous DSBs, we costained NONO with the p53-binding protein 1 (53BP1) or γH2A.X (Supplementary Figure S4). Whilst 53BP1 and γH2A.X clearly colocalised, no obvious colocalisation of NONO with the DSB markers could be observed upon etoposide treatment. We conclude that the accumulation of NONO at DSB foci is modest.

### 3.3 NONO-APEX2-HA Accumulates in the Nucleolus of DNA-Damaged Cells

Next, we tested the expression and localisation of NONO-APEX2-HA in response to DNA damage. We first monitored the size and expression level of NONO-APEX2-HA by immunoblotting (Figure 2D). Samples from U2OS:NONO-APEX2-HA, but not wild type cells, displayed HA antibody reactivity at 86 kDa irrespective of DNA damage, which was confirmed by 6-8-fold stronger γH2A.X signals. The NONO antibody generated a 55 kDa band in all conditions and an additional 86 kDa band in samples from U2OS:NONO-APEX2-HA cells. To assess the NONO-APEX2-HA localisation, we used the HA antibody in confocal microscopy in combination with NPM1 staining. The HA antibody stained nucleoplasmic in unperturbed cells, but displayed additional nucleolar reactivity in 50–60% of DNA-damaged cells (Figure 2E). RGB profiler measurements revealed elevated colocalisation of HA and NPM1 signals in the presence of etoposide (Figure 2F). We conclude that NONO-APEX2-HA expresses irrespective of DNA damage and localises comparable to endogenous NONO.

### 3.4 NONO-APEX2-HA Interacts with Components of Paraspeckles

**FIGURE 3:**
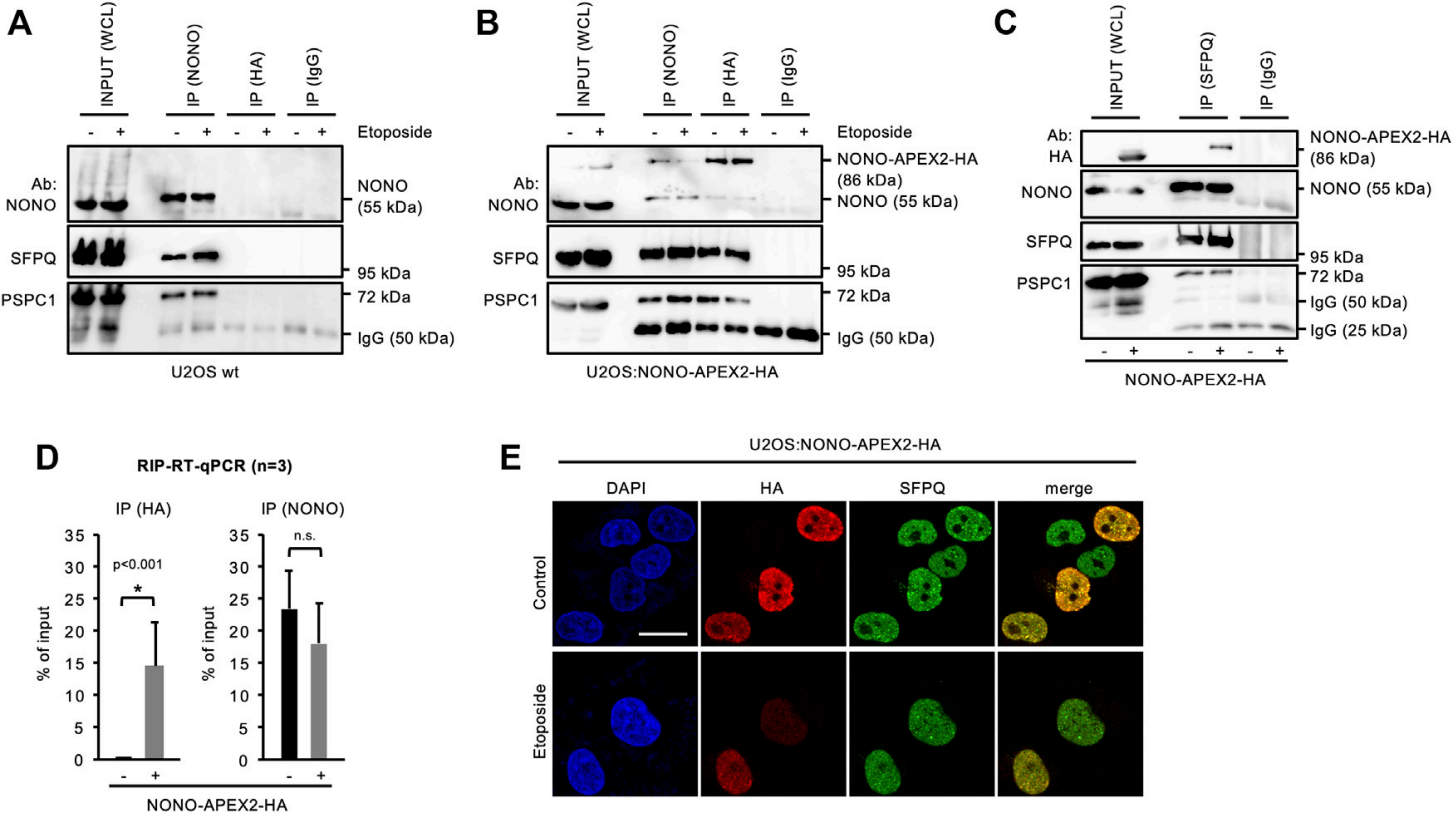
Interaction of NONO-APEX2-HA with SFPQ and PSPC1. **(A–C)** Immunoblots detecting NONO, NONO-APEX2-HA, SFPQ and PSPC1 with or without (+/-) Etoposide (20 μM, 2 h) in whole cell lysates (WCL) or upon immunoprecipitation (IP) from wild type (wt) U2OS **(A)** or U2OS:NONO-APEX2-HA cells **(B)** or from wt U2OS (-) or U2OS:NONO-APEX2-HA (+) cells **(C)**. Ab, antibody; IgG, immunoglobulin chain. **(D)** Reverse-transcriptase quantitative PCR (RT-qPCR) with NEAT1-selective primer upon RNA immunoprecipitation (RIP) from WCL of wt (-) or U2OS:NONO-APEX2-HA (+) cells with the HA antibody (left) or the NONO antibody (right). Asterisk, *p* < 0.001 (Student’s t-test); n.s., not significant. **(E)** Confocal imaging of NONO-APEX2-HA and SFPQ ± Etoposide (20 μM, 2 h). Representative cells are shown. DAPI, 4‘,6-diamidino-2-phenylindol; scale bar, 10 µm.

NONO forms complexes with the splicing factor proline and glutamine rich (SFPQ) and the paraspeckle component 1 (PSPC1) (Knott et al., 2016). Thus, we asked if NONO-APEX2-HA associates with paraspeckle proteins. We immunoprecipitated NONO from wild type U2OS cells and detected comparable amounts of NONO, SFPQ and PSPC1, irrespective of DNA damage (Figure 3A). Next, we immunoprecipitated NONO-APEX2-HA using NONO or HA antibodies (Figure 3B). Again, we coenriched paraspeckle components irrespective of DNA damage. Strikingly, we detected an additional 86 kDa band that was clearly enriched after immunoprecipitation with the HA antibody. Further, we immunoselected SFPQ from U2OS wild type and U2OS:NONO-APEX2-HA lysates (Figure 3C). The SFPQ antibody coenriched paraspeckles proteins from both lysates, but NONO-APEX2-HA from the U2OS:NONO-APEX2-HA lysate only. We also assessed if NONO-APEX2-HA associates with the nuclear enriched assembly transcript 1 (NEAT1). The long non-coding RNA NEAT1 is essential for the formation of paraspeckles and a cognate substrate of NONO (Shav-Tal and Zipori, 2002; Chen and Carmichael, 2009; Clemson et al., 2009; Sasaki et al., 2009; Knott et al., 2016). We immunoprecipitated NONO-APEX2-HA with the HA antibody and quantified the level of coenriched NEAT1 transcripts by RT-qPCR and could detect significantly enriched levels of NEAT1 upon HA immunoselection from lysates of U2OS:NONO-APEX2-HA, but not wild type cells (Figure 3D, left). Next, we repeated the immunoprecipitation with the NONO antibody. Strikingly, we coenriched similar amounts of NEAT1 from wild type and U2OS:NONO-APEX2-HA cells (Figure 3D, right). Further, we compared the subcellular localisation of NONO-APEX2-HA with paraspeckle proteins and DSB markers (Figure 3E and Supplementary Figure S5). Indeed, cells that stained positive for NONO-APEX2-HA displayed a strong colocalisation with NONO, SFPQ and PSPC1, but neither with 53BP1 nor γH2A.X. We conclude that NONO-APEX2-HA associates with paraspeckle components and the lncRNA NEAT1.

### 3.5 NONO-APEX2-HA Is Capable of *in vivo* Proximity Labeling

**FIGURE 4:**
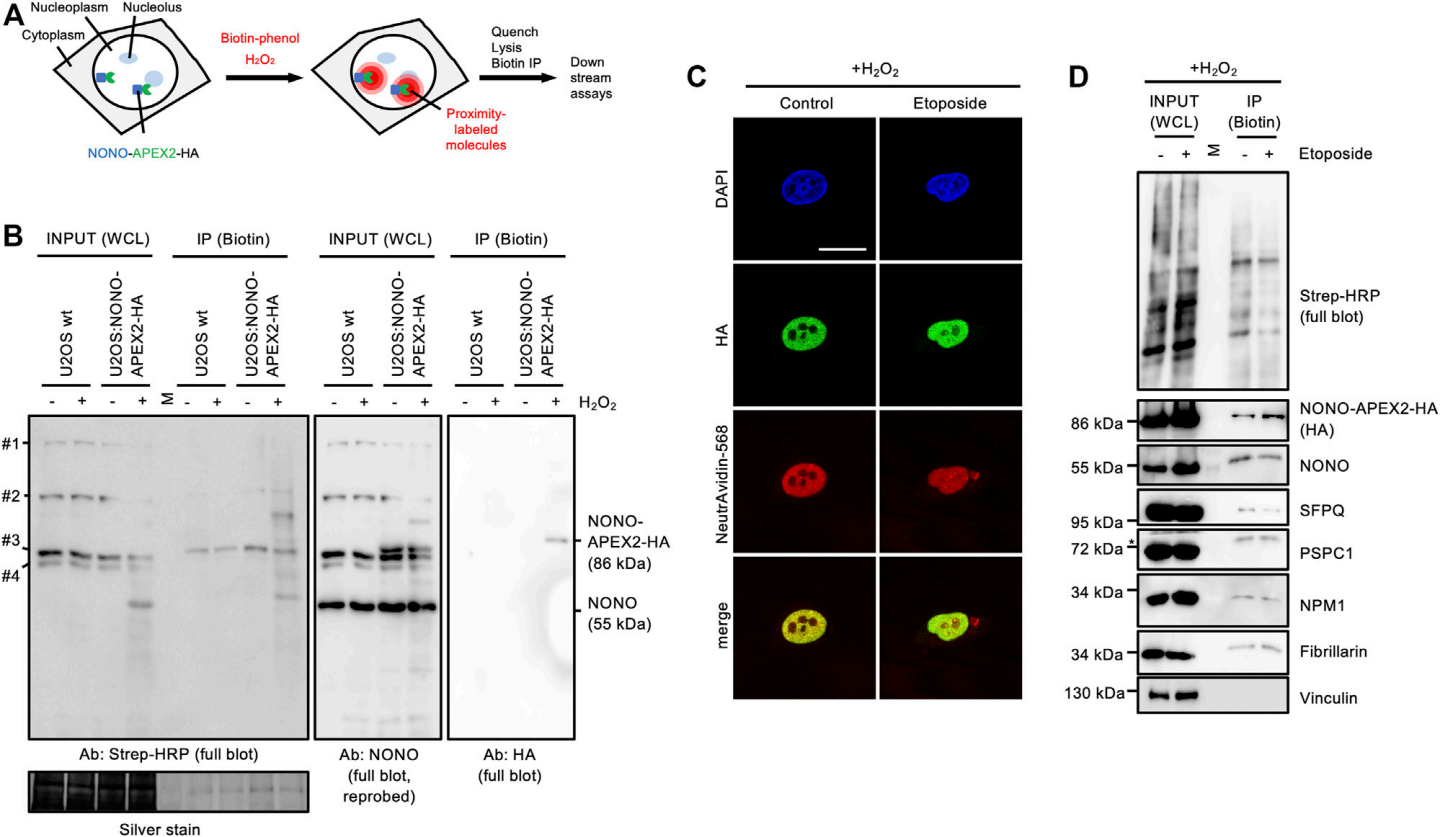
*In vivo* proximity labeling in U2OS:NONO-APEX2-HA cells. **(A)** Workflow of *in vivo* proximity labeling including expression of the APEX2 fusion protein, addition of biotin-phenol and hydrogen peroxide (H_2_O_2_), quenching of reaction, lysis and immunoprecipitation of biotinylated proteins (Biotin IP). **(B)** Immunoblots detecting biotinylated proteins (Strep-HRP), NONO and NONO-APEX2-HA from whole cell lysates (WCL) or upon IP from wt U2OS or U2OS:NONO-APEX2-HA cells treated with or without (+/-) H_2_O_2_. #1, CoA carboxylase (280 kDa); #2, Pyruvate carboxylase (128 kDa); #3, Propionyl-CoA carboxylase (74 kDa); #4, β-methylcrotonyl-CoA carboxylase (72 kDa); Ab, antibody; Strep-HRP, streptavidin-horse radish peroxidase; M, protein standard; silver stain, loading control. **(C)** Confocal imaging of NONO-APEX2-HA and biotin (NeutrAvidin-568) +/- Etoposide (20 μM, 2 h). Representative images are shown. DAPI, 4‘,6-diamidino-2-phenylindol; scale bar, 10 µm. **(D)** Immunoblots detecting biotin (Strep-HRP) or indicated proteins from WCL or after IP ± Etoposide (20 μM, 2 h). M, protein standard; asterisk, cut membrane.

Next, we wished to test if NONO-APEX2-HA is catalytically active. The human genome encodes naturally biotinylated enzymes (Knowles, 1989). We first asked if these are detectable in wild type U2OS cells. Using a horse radish peroxidase-conjugated streptavidin probe (Strep-HRP) on immunoblots, we observed four bands that migrated at predicted sizes, but were not detectable upon NONO immunoselection (Supplementary Figure S6A). APEX2 uses hydrogen peroxide (H_2_O_2_) to generate reactive biotin-phenol radicals that conjugate with proximal amino acids and transcripts (Fazal et al., 2019; Hung et al., 2016; Lam et al., 2015) (Figure 4A). To test labeling kinetics, we incubated U2OS:NONO-APEX2-HA cells with 1 mM H_2_O_2_ (Supplementary Figure S6B). We observed a smear of Strep-HRP signals in the inputs and upon biotin immunoprecipitation in the presence of H_2_O_2_, but only naturally biotinylated proteins without H_2_O_2_. Reassuringly, the HA antibody displayed a single 86 kDa band upon biotin immunoprecipitation, most clearly after a brief incubation with H_2_O_2_. We repeated the labeling and included wild type cells as control (Figure 4B). Again, Strep-HRP displayed naturally biotinylated proteins in all input conditions, whereas additional biotinylated proteins were only observed in samples from U2OS:NONO-APEX2-HA cells that were incubated with H_2_O_2_. We reprobed the Strep-HRP blot with the NONO antibody and detected NONO at 55 kDa in all conditions and NONO-APEX2-HA at 86 kDa specifically in samples from U2OS:NONO-APEX2-HA cells irrespective of H_2_O_2_ treatment. Upon biotin immunoprecipitation, however, the HA antibody stained NONO-APEX2-HA only in the presence of H_2_O_2_. Next, we assessed biotinylation *in vivo*. We tested if biotin could be traced as pan-nuclear signal and costained NONO-APEX2-HA with biotin using a NeutrAvidin-568 probe (Figure 4C). Indeed, NeutrAvidin-568 displayed strong colocalisation with HA in the nucleoplasm, but not the cytoplasm of unperturbed cells. In the presence of etoposide, pan-nuclear NeutrAvidin-568 staining could be observed. Biotin-phenol and H_2_O_2_ raise some concerns about toxicity (Qin et al., 2021; Weissinger et al., 2021). Thus, we monitored the onset of DNA damage by APEX2 substrates in U2OS:NONO-APEX2-HA cells. We assessed the levels of the tumour suppressor protein p53, γH2A.X, serine-1981-phosphorylated ataxia telangiectasia mutated (pATM) kinase and its phospho-substrates by immunoblotting (Supplementary Figure S6C). We observed little induction of DNA damage markers by the APEX2 substrates, but strong induction of DNA damage by etoposide, which was not further elevated by the combination of APEX2 substrates and etoposide. This suggests that the bulk of DNA damage is triggered by etoposide, but not by APEX2 substrates. Finally, we tested if NONO-APEX2-HA biotinylates distinct proteins. We performed proximity labeling and probed for paraspeckle components (NONO, SFPQ, PSPC1), nucleolar proteins (NPM1, Fibrillarin), and cytoplasmic Vinculin on immunoblots (Figure 4D). Indeed, we detected the nuclear and nucleolar proteins, but not Vinculin, upon biotin immunoprecipitation irrespective of DNA damage and dependent on the presence of H_2_O_2_ (Supplementary Figure S6D). We conclude that NONO-APEX2-HA biotinylates proteins *in vivo*.

## 4 Discussion

Proximity interactomes are often prone to a high number of false positive candidates due to random spatial association with the protein of interest. Thus, enzyme tags for improved proximity ligation are steadily developed. A range of biotin ligases such as BirA*, BASU or TurboID offer both advantages and disadvantages compared to APEX2 (Rees et al., 2015; Kim and Roux, 2016; Trinkle-Mulcahy, 2019; Ummethum and Hamperl, 2020). We describe a novel U2OS cell line that expresses a stable NONO-APEX2-HA fusion protein suitable for *in vivo* proximity labeling. The subcellular localisation of NONO-APEX2-HA is comparable to endogenous NONO and phenocopies elevated nucleolar localisation in DNA-damaged cells. NONO-APEX2-HA associates with paraspeckle proteins and biotinylates them *in vivo*. We opted for APEX2, as it allows fast labeling. APEX2 comprises a 10 times shorter labeling time (1 min compared to at least 10 min with TurboID) and produces biotin-phenol radicals with extremely short half live (<1 ms compared to minutes for the biotin ligase product biotinoyl-5′-AMP) (Rhee et al., 2013; Hung et al., 2016). Unlike biotin ligases, APEX2 targets various amino acids and produces a labeling gradient. Biotin ligases, in contrast, are biased for proteins with intrinsically disordered regions, which are often enriched in lysines (Minde et al., 2020). These features are relevant for NONO, an abundant RBP with putative roles in the DDR. As DSB sensing occurs within seconds, we aimed for a fast proximity ligation system with a tolerable amount of collateral damage. Moreover, the interactome of NEAT1 has recently been assessed by APEX2 via hybridisation dependent proximity ligation (Mamontova et al., 2022; Yap et al., 2022). Thus, future interactome studies using U2OS:NONO-APEX2-HA cells may benefit from cross validation by the APEX2-derived NEAT1 interactome.

We noticed a certain variability in NONO stainings, ranging from pan-nuclear to more prominently nucleolar. The nucleolus is a sensor of cellular stress and disintegrates in the presence of various drugs, including etoposide, which is reflected by nucleoplasmic translocations of nucleolar proteins like NPM1 (Rubbi and Milner, 2003; Kurki et al., 2004; Shav-Tal et al., 2005; Burger et al., 2010; Burger and Eick, 2013). It is possible that etoposide-induced nucleolar stress contributes to the variability in NONO imaging. However, we titrated etoposide previously and reported nucleolar stress at higher doses (Burger et al., 2010). As a subset of NONO colocalises with NPM1 in DNA-damaged cells, etoposide may have minor impact on nucleolar integrity. We predicted a DNA damage-inducible biotinylation of a subset of nucleolar proteins by NONO-APEX2-HA, but failed to detect changes on immunoblots for the tested candidates. We speculate that a relatively small subset of the NONO pool enriches in the nucleolus in DNA-damaged cells. Likewise, NONO does not fully colocalise with nucleolar markers in non-disintegrated nucleoli in response to etoposide. NONO may be differentially responsive to the two major DSB repair pathways homologous recombination (HR) and non-homologous end-joining (NHEJ). The nucleolar accumulation of NONO may predominantly be triggered by HR or NHEJ or occur cell cycle specific.

It is currently unclear, whether the nucleolar NONO localisation is linked to the DDR. The nucleolus tethers numerous non-nucleolar proteins in response to cellular stress (Audas et al., 2012; Chen and Stark, 2019; Frottin et al., 2019; Mamontova et al., 2021). Upon heat shock, for example, >200 proteins associate with nucleolar NPM1, including NONO. The nucleolus may support the DDR by monitoring protein integrity. NONO may also transit through the nucleolus in analogy to the tumour suppressor p53, which is modified in the nucleolus (Boyd et al., 2011). Other RBPs have also been reported in the nucleolus upon stress (Martinez-Macias et al., 2019; Chen et al., 2020). The paraspeckle components SFPQ and fused in sarcoma (FUS) strongly accumulate in the nucleolus in response to UV irradiation and incubation with the topoisomerase inhibitor camptothecin, respectively. Interestingly, FUS enriches at sites of nucleolar transcription in the ribosomal DNA cluster, but reduces its binding to sites of RNAPII transcription, suggesting a role in balancing the RNA metabolism in the DDR. Moreover, many RBPs promote RNAPII transcription by stimulating phase separation in the nucleoplasm (Shao et al., 2021). The nucleolar accumulation of NONO and other RBPs may thus reflect a mechanism to mitigate stress. Overall, our data indicate that U2OS:NONO-APEX2-HA cells facilitate specific and sensitive studies on NONO interactome dynamics.

## Data Availability

The original contributions presented in the study are included in the article/Supplementary Material, further inquiries can be directed to the corresponding author.
