# Supplementary material for: In vivo Proximity Labeling of Nuclear and Nucleolar Proteins by a Stably Expressed, DNA Damage-Responsive NONO-APEX2 Fusion Protein
Source: Front Mol Biosci. 2022 Jun 6;9:914873. doi: 10.3389/fmolb.2022.914873 (PMC9207311; doi:10.3389/fmolb.2022.914873)
Supplement: Supplementary file 1 [file DataSheet1.PDF]

# Supplementary Material

1 Supplementary Figures

2 Supplementary Tables

# Supplementary Figure 1

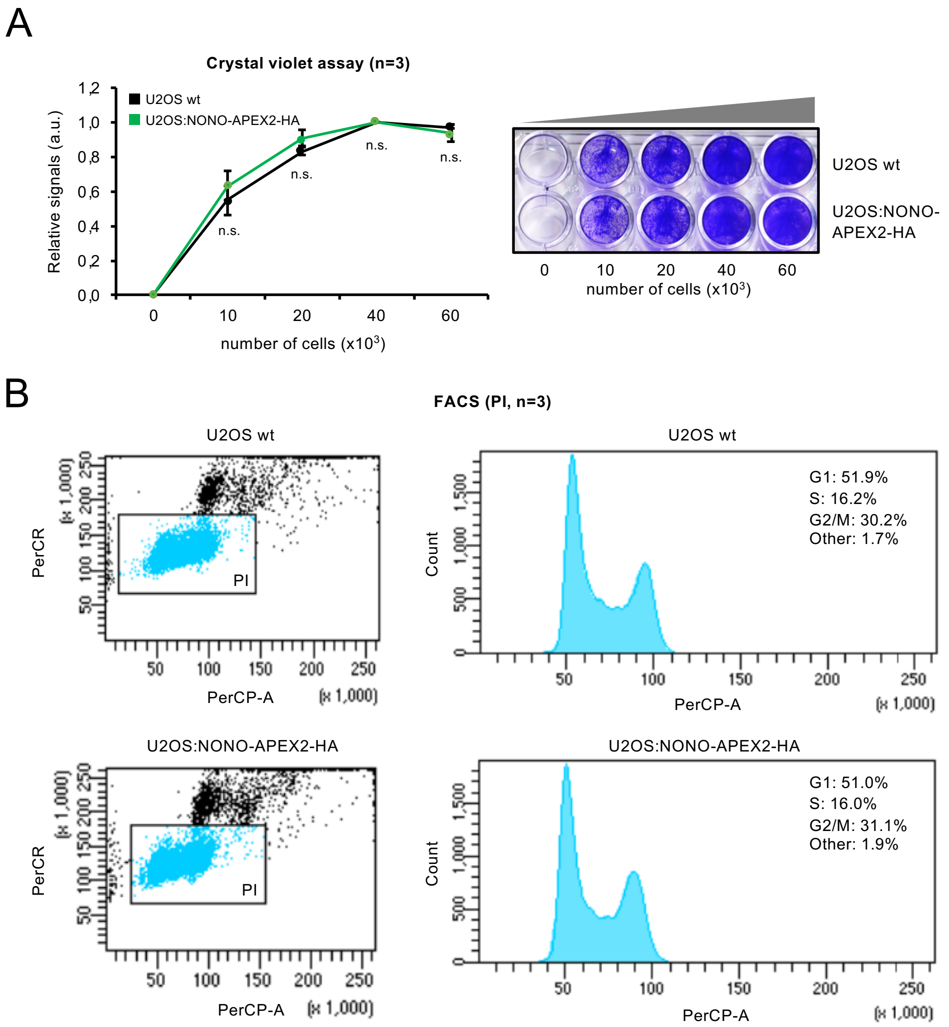


Supplementary Figure 1. **Proliferation and cell cycle analysis of U2OS:NONO-APEX2-HA cells.** (**A**) Crystal violet staining of wild type (wt) U2OS or U2OS:NONO-APEX2-HA cells are plotted as relative signals (left). Signal intensities were quantified by ImageJ and set to 1 for 40x10³ cells. a.u., arbitrary units; n.s., not significant. Significance was analysed by a Student's t-test. Scan of representative staining (right). (**B**) Cell cycle analysis by fluorescence-activated cell sorting (FACS). Gating of propidium iodide (PI)-stained wild type (wt) U2OS or U2OS:NONO-APEX2-HA cells for viable, non-duplet cells (left) and stratification for cell cycle phase (right). PerCP-A/ PerCR, forward/sideward scatter. A representative experiment is shown.

# Supplementary Figure 2

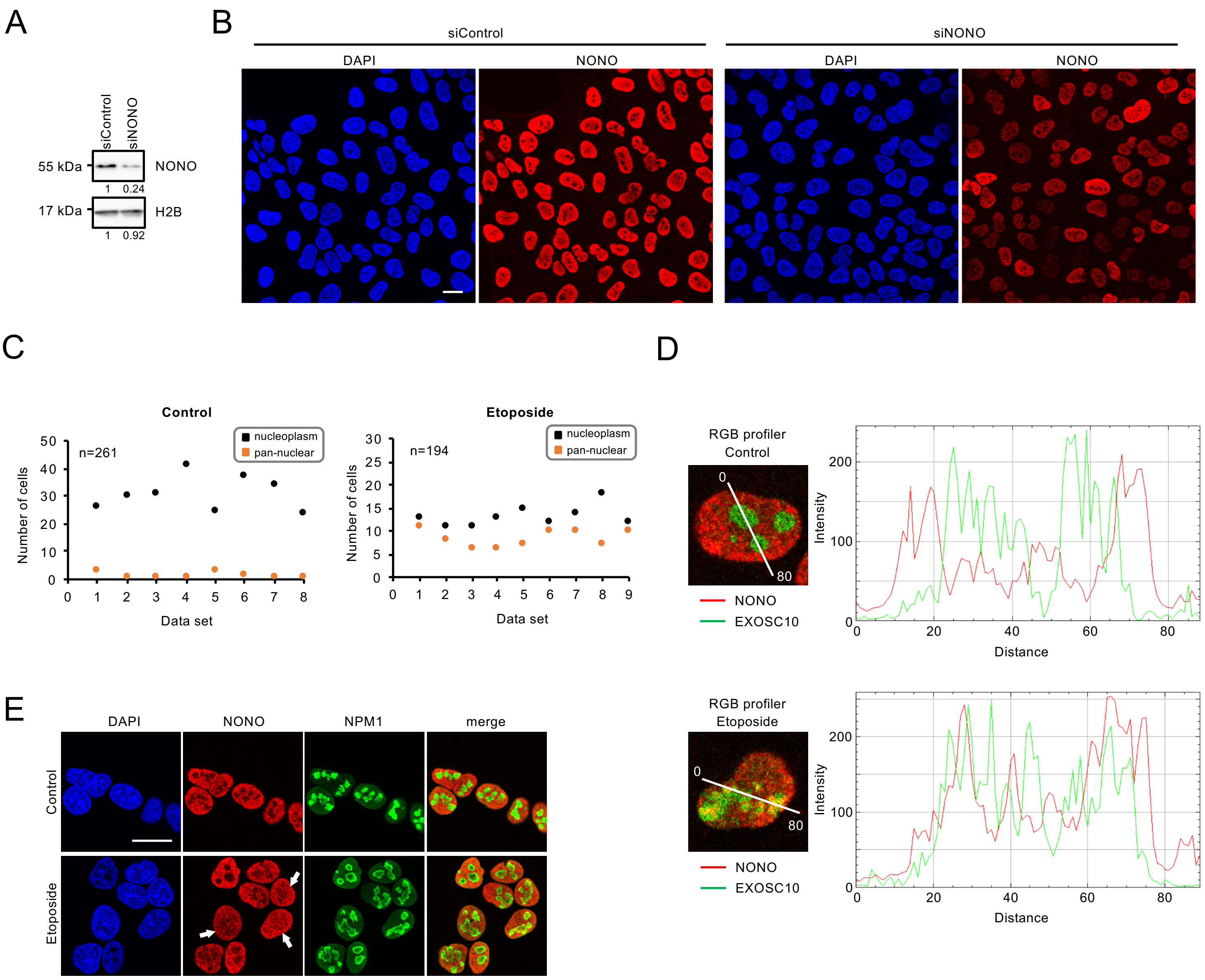


Supplementary Figure 2. **NONO antibody validation and assessment of subcellular NONO localisation in U2OS cells.** (**A**) Immunoblots detecting NONO upon transfection of small-interfering (si)RNA smart pools. H2B, histone 2B; loading control. Immunoblots were quantified by ImageJ. (**B**) Confocal imaging of NONO upon transfection of siRNA targeting NONO (siNONO) or non-targeting control (siControl). (**C**) Stratification of NONO subcellular localisation shows number of cells that display NONO staining in the nucleoplasm (black) or as pan-nuclear (orange). (**D**) RGB profiler plots for zoomed panels from Figure 2B. (**E**) Confocal imaging of NONO and Nucleophosmin 1 (NPM1) in the absence or the presence of Etoposide. Representative cells are shown. DAPI, 4‘,6-diamidino-2-phenylindol; scale bar, 10 µm; white arrowheads, pan-nuclear NONO staining.

# Supplementary Figure 3

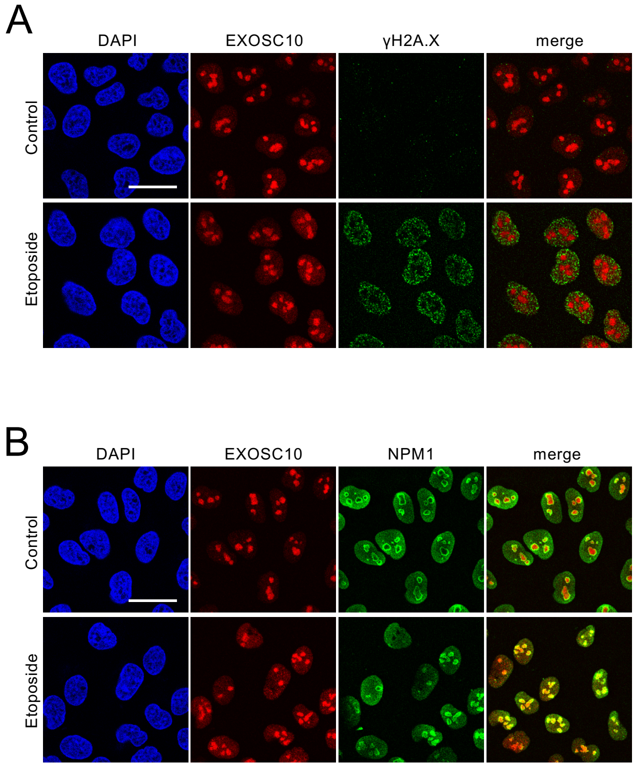


Supplementary Figure 3. **Preservation of nucleolar integrity upon short-term treatment with Etoposide in U2OS cells.** (**A**) Confocal imaging of EXOSC10 and γH2A.X in the absence or the presence of Etoposide. (**B**) Confocal imaging of EXOSC10 and NPM1 in the absence or the presence of Etoposide. Representative cells are shown. DAPI, 4',6-diamidino-2-phenylindol; scale bar, 10 µm.

# Supplementary Figure 4

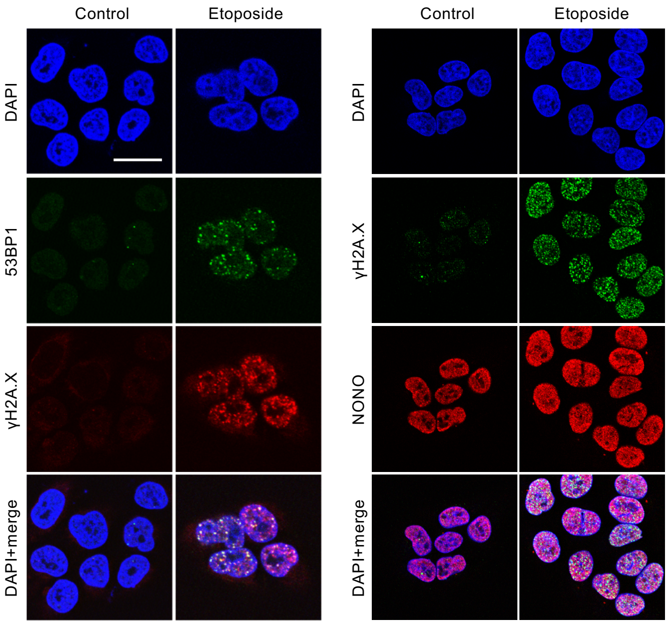


Supplementary Figure 4. **Colocalisation of γH2A.X with 53BP1, but not NONO in U2OS cells.** Confocal imaging of the p53-binding protein 1 (53BP1) and γH2A.X (left) or γH2A.X and NONO (right) in the absence or the presence of Etoposide. Representative cells are shown. DAPI, 4',6-diamidino-2-phenylindol; 4OHT, 4-hydroxy-tamoxifen; scale bar, 10 µm.

# Supplementary Figure 5

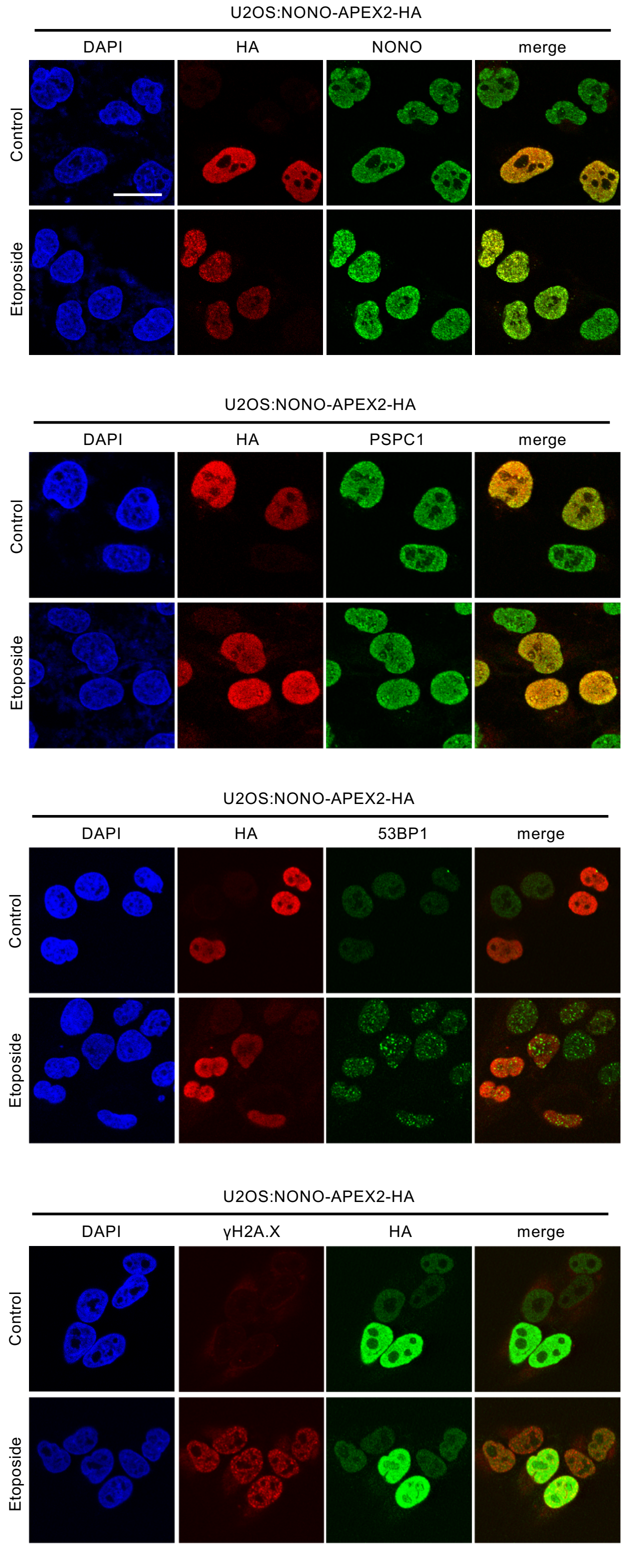


Supplementary Figure 5. **Colocalisation of NONO-APEX2-HA with NONO and PSPC1, but not 53BP1 and γH2A.X in U2OS:NONO-APEX2-HA cells.** Confocal imaging of NONO-APEX2-HA with NONO, PSPC1, 53BP1 or γH2A.X (top to bottom) in the absence or the presence of Etoposide. Representative cells are shown. DAPI, 4',6-diamidino-2-phenylindol; scale bar, 10 µm.

# Supplementary Figure 6

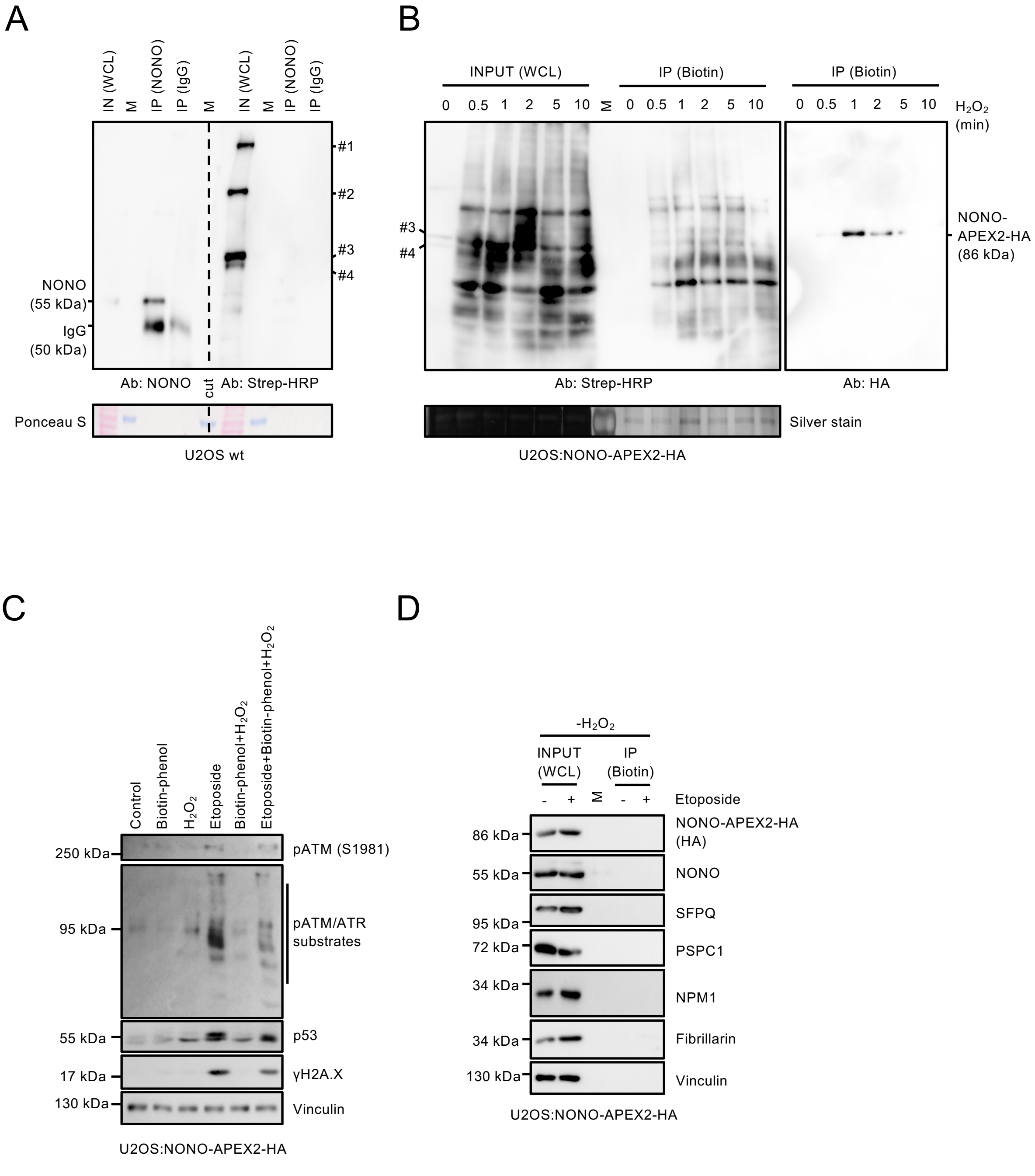


Supplementary Figure 6. **Quality control for *in vivo* proximity labeling.** (**A**) Immunoblot detecting NONO or biotinylated proteins (Strep-HRP) from whole cell lysates (WCL) or upon immunoprecipitation (IP) from U2OS wild type (wt) cells. #1, CoA carboxylase (280 kDa); #2, Pyruvate carboxylase (128 kDa); #3, Propionyl-CoA carboxylase (74 kDa); #4, β-methylcrotonyl-CoA carboxylase (72 kDa); Ab, antibody; Strep-HRP, streptavidin-horse radish peroxidase; M, protein standard; Ponceau S, loading control; IgG; immunoglobulin; dashed line, cut membrane. (**B**) Immunoblots detecting biotinylated proteins (Strep-HRP) or NONO-APEX2-HA from WCL or upon IP from U2OS:NONO-APEX2-HA cells treated in a hydrogen peroxide ($H_2O_2$) time kinetic. Silver stain, loading control. (**C**) Immunoblots detecting Ser1981-phosphorylated ataxia telangiectasia mutated (pATM), substrates of ATM/ataxia telangiectasia mutated-related (pATM/ATR substrates), p53 and γH2A.X from WCL after incubation with Biotin-phenol (0.5 mM, 30 min), $H_2O_2$ (1 mM, 1 min), Etoposide (20 μM, 2 h) or combinations thereof. Vinculin, loading control. (**D**) Immunoblots detecting indicated proteins from WCL or after IP with or without Etoposide (20 μM, 2 h) in the absence of $H_2O_2$.

# Supplementary Figure 7

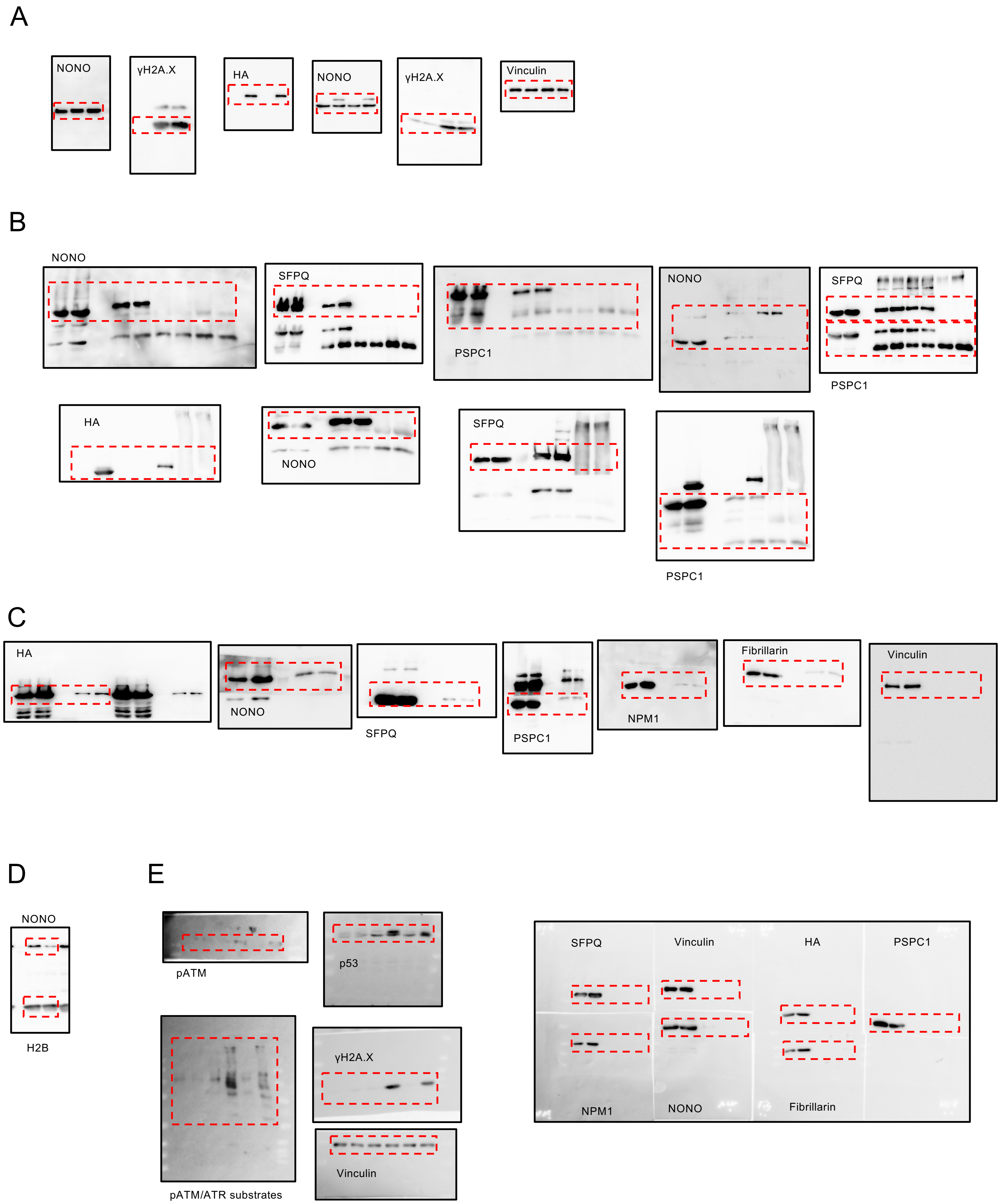


Supplementary Figure 7. **Panels of uncropped images of immunoblots.** (**A**) Uncropped images from Figure 2. (**B**) Uncropped images from Figure 3. (**C**) Uncropped images from Figure 4. (**D**) Uncropped images from Supplementary Figure 2. (**E**) Uncropped images from Supplementary Figure 6. Broken red boxes indicate cropped regions.

# Supplementary Table 1

| Primer | Sequence (5’-3’) |
|---|---|
| APEX2 Entry-fwd | ccTTAATTAAcgACGCGTGGTGGTGGTTCTggaaagtcttac |
| APEX2 Entry-rev | acactagtctaGGCGTAATCTGGAACATCGTATGGGTAggcatcagcaaa |
| NONO PCR-fwd | ccTTAATTAAccaccatgcagagtaataaaacttttaacttggagaagca |
| NONO PCR-rev | cgcgACGCGTgtatcggcgacgtttgtttggg |
| NEAT1 RT-qPCR-fwd | TTGACCAACGCTTTATTTTC |
| NEAT1 RT-qPCR-rev | TTACCAACAATACCGACTCC |

Supplementary Table 1. **Primers for PCR cloning and RT-qPCR used in this study.** The APEX2 Entry-fwd primer contains a linker (GGTGGTGGTTCT), the APEX2 Entry-rev primer contains a stop codon and an HA tag (ctaGGCGTAATCTGGAACATCGTATGGGTA). The NONO PCR fwd primer contains a Kozak sequence and a start codon (ccaccatg). The NEAT1 primers target both short and long NEAT1 isoforms.

# Supplementary Table 2

| Antibody | Source |
|---|---|
| anti-NONO | Proteintech, 11058-1-AP |
| anti-EXOSC10 | Abcam, ab50558 |
| anti-γH2A.X (S139) | Merck Millipore, 05-636 |
| anti-γH2A.X (S139) | Cell Signaling Tech., 2577 |
| anti-NPM1 | Abcam, ab10530 |
| anti-HA tag | Abcam, ab9110 |
| anti-HA tag | Biolegend, 901501 |
| anti-Fibrillarin | Abcam, ab911 |
| anti-Vinculin | Sigma, V9131 |
| anti-SFPQ | Abcam, ab177149 |
| anti-PSPC1 | Proteintech, 16714-1-AP |
| anti-H2B | Abcam, ab1790 |
| anti-53BP1 | Novus, NB100-304 |
| anti-p53 | Santa Cruz, sc-126, DO-1 |
| anti-phospho-ATM (S1981) | Abcam, ab81292 |
| anti-phospho-ATM/ATR Substrate (S*Q) | Cell Signaling Tech., 9607 |
| anti-IgG control | Proteintech, 30000-0-AP |
| anti-Streptavidin-HRP | Cytivia, RPN123 |
| anti-rabbit-IgG-HRP | Cytivia, NA934 |
| anti-mouse-IgG-HRP | Cytivia, NA931 |
| Alexa fluor 568-goat-anti-rabbit-IgG | Invitrogen, A11036 |
| Alexa fluor 488-goat-anti-mouse-IgG | Invitrogen, A11001 |
| Alexa fluor 546-donkey-anti-mouse-IgG | Invitrogen, A10036 |
| Alexa fluor 488-donkey-anti-mouse-IgG | Invitrogen; A21206 |
| Avidin-neutravidin-tetramethylrhodamine (NeutrAvidin-568) | Invitrogen, A6373 |

Supplementary Table 2. **Primary and secondary antibodies used in this study.**
